# Antibody epitope profiling of the KSHV LANA protein using VirScan

**DOI:** 10.1371/journal.ppat.1011033

**Published:** 2022-12-19

**Authors:** Sydney J. Bennett, Dicle Yalcin, Sara R. Privatt, Owen Ngalamika, Salum J. Lidenge, John T. West, Charles Wood

**Affiliations:** 1 School of Biological Sciences, University of Nebraska-Lincoln, Lincoln, Nebraska, United States of America; 2 Department of Interdisciplinary Oncology, Stanley S. Scott Cancer Center, Louisiana State University Health Sciences Center, New Orleans, Louisiana, United States of America; 3 Dermatology and Venereology Section, University Teaching Hospital, University of Zambia School of Medicine, Lusaka, Zambia; 4 Ocean Road Cancer Institute, Dar es Salaam, Tanzania; 5 Department of Clinical Oncology, Muhimbili University of Health and Allied Sciences, Dar es Salaam, Tanzania; Oregon Health and Science University, UNITED STATES

## Abstract

The humoral antibody response against Kaposi sarcoma-associated herpesvirus (KSHV) in infected individuals has been characterized demonstrating the latency-associated nuclear antigen (LANA) as the most antigenic KSHV protein. Despite the antigenicity of the protein, specific LANA epitopes have not been systematically characterized. Here, we utilized a bacteriophage T7 library, which displays 56-amino acid KSHV LANA peptides with 28-amino acid overlap (VirScan), to define those epitopes in LANA targeted by antibodies from a cohort of 62 sub-Saharan African Kaposi sarcoma (KS) patients and 22 KSHV-infected asymptomatic controls. Intra- and inter-patient breadth and magnitude of the anti-LANA responses were quantified at the peptide and amino acid levels. From these data, we derived a detailed epitope annotation of the entire LANA protein, with a high-resolution focus on the N- and C-termini. Overall, the central repeat region was highly antigenic, but the responses to this region could not be confidently mapped due to its high variability. The highly conserved N-terminus was targeted with low breadth and magnitude. In a minority of individuals, antibodies specific to the nuclear localization sequence and a portion of the proline-rich regions of the N-terminus were evident. In contrast, the first half of the conserved C-terminal domain was consistently targeted with high magnitude. Unfortunately, this region was not included in LANA partial C-terminal crystal structures, however, it was predicted to adopt predominantly random-coil structure. Coupled with functional and secondary structure domain predictions, VirScan revealed fine resolution epitope mapping of the N- and C-terminal domains of LANA that is consistent with previous antigenicity studies and may prove useful to correlate KSHV humoral immunity with pathogenesis.

## Author summary

The incidence of Kaposi sarcoma (KS) coincides with high prevalence of Kaposi sarcoma-associated herpesvirus (KSHV) infection, particularly in sub-Saharan Africa, Peru, and the Mediterranean. KSHV infection typically occurs during childhood and enters latency typified by the expression of latency-associated nuclear antigen (LANA). Despite recognition that LANA is highly antigenic, no previous study has defined the targeted epitopes. Our study presents the first high-resolution epitope map of LANA in KS patients and asymptomatic KSHV-infected individuals. High-throughput phage display, VirScan, was leveraged to elucidate epitopes across the LANA protein and compare them between KS and asymptomatic groups. A 24-amino acid peptide was found to be highly antigenic in both groups, regardless of co-infection with human immunodeficiency virus (HIV). In KS patients, HIV co-infection was associated with a lower response to this epitope, but HIV co-infection had little to no effect in the absence of cancer. Further studies of the identified epitope will determine whether the epitope has therapeutic, diagnostic, or viral decoy potential. The application of our novel findings will be instrumental when applied to the entire KSHV proteome in designing effective vaccines for KSHV infection and therapeutics to prevent progression to KS.

## Introduction

Kaposi sarcoma-associated herpesvirus (KSHV) is the causative agent of Kaposi sarcoma (KS), which occurs at high incidence in sub-Saharan Africa (SSA), and a number of other geographic locations [1]. KS incidence is higher among individuals co-infected with Human Immunodeficiency Virus-1 (HIV; Epidemic KS) compared to HIV^-^ individuals (Endemic KS), due to acquired immunodeficiency syndrome (AIDS)-induced immunosuppression. Latency-associated nuclear antigen (LANA) is a 1,162 amino acid (230kDa) protein encoded by the KSHV ORF73 gene (NC_009333.1). LANA is consistently and abundantly detected in KSHV-infected KS tumor cells, KSHV primary effusion lymphoma cells, multicentric Castleman’s disease B cells, and latently infected cells *in vitro*. LANA is a multifunctional oncoprotein that plays a role in KSHV-mediated tumorigenesis through its manipulation of cell cycle machinery and deregulation of tumor suppressor pathways. Further, LANA is highly antigenic, and along with the lytic glycoprotein K8.1, its serological detection is considered the gold standard for diagnosis of KSHV infection in latency and lytic replication, respectively [2]. When detected, LANA exhibits a punctate nuclear staining pattern in infected or tumor cells that is diagnostic of KSHV etiology.

Structurally, LANA has conserved amino (N-) and carboxyl (C-) terminal regions, interspaced by an extensive, highly variable, and acidic series of repeats. The N-terminus, also known as the chromosome-binding motif (CBM), has been shown to tether the viral episome to host chromatin by interacting with histones H2A and H2B. This region plays a central role in promoting KSHV episomal persistence by coordinating episome duplication and partitioning with cellular replication [3]. This coordination also helps keep KSHV genome burden and antigen low in most infected cells. On the other hand, the C-terminus is commonly referred to as the KSHV DNA-binding domain (DBD) since it binds directly to the conserved terminal repeats (TR) in the KSHV episome to maintain viral genomic stability. Therefore, LANA constitutes a link between cellular chromatin and the KSHV episome. The C-terminal domain has a predicted hydrophobic 3-dimensional core structure. Mutagenesis of the hydrophobic residues in this tetramer interface reveals that they are essential for higher order oligomerization of LANA, which imparts the cooperativity necessary for DNA-protein complex formation and contributes to genome stability, persistence, and replication [4].

Seroepidemiological studies using protein- and infected cell-based assays to detect and quantify KSHV seroprevalence and humoral antibody (Ab) response have employed immunofluorescence assays (IFA), luciferase immunoprecipitation systems (LIPS), enzyme-linked immunosorbent assays (ELISA), and protein bead-arrays, each varying in specificity and sensitivity, yet overall contributing major advancements to Ab response evaluation and diagnosis of KSHV infection. Phage display technologies have recently been developed to present large libraries of potential peptide epitopes in tiled arrays to facilitate mapping of all potential Ab recognition sites that other immunoassays cannot do readily or systematically. For example, it is possible to profile Ab repertoires against entire proteomes without having to purify the target proteins or analyze each peptide individually. Moreover, individual- and group-specific Ab responses can be defined comparatively between those with and without disease or with and without an infection.

Although extensively studied and demonstrated to be highly antigenic, LANA epitopes have rarely been investigated and never using a high-resolution tiled phage display system. In this study, we mapped epitopes of the KSHV LANA protein using phage display and immunoprecipitation followed by barcoding and high-throughput sequencing (PhIP-Seq). LANA peptides were displayed on T7-bacteriophage as a library of 56-amino acid tiles, each with a 28-amino acid overlap (VirScan) [5,6]. We quantified the recognition of the peptide epitopes in LANA and comparatively assessed the breadth and magnitude of the anti-LANA response within and between 16 HIV^-^ Endemic KS (EnKS) patients, 46 HIV^+^ Epidemic KS (EpKS) patients, and 22 KSHV-infected individuals without KS (asymptomatic) from SSA.

## Results

### Phage library and cohort characteristics

**Fig 1:**
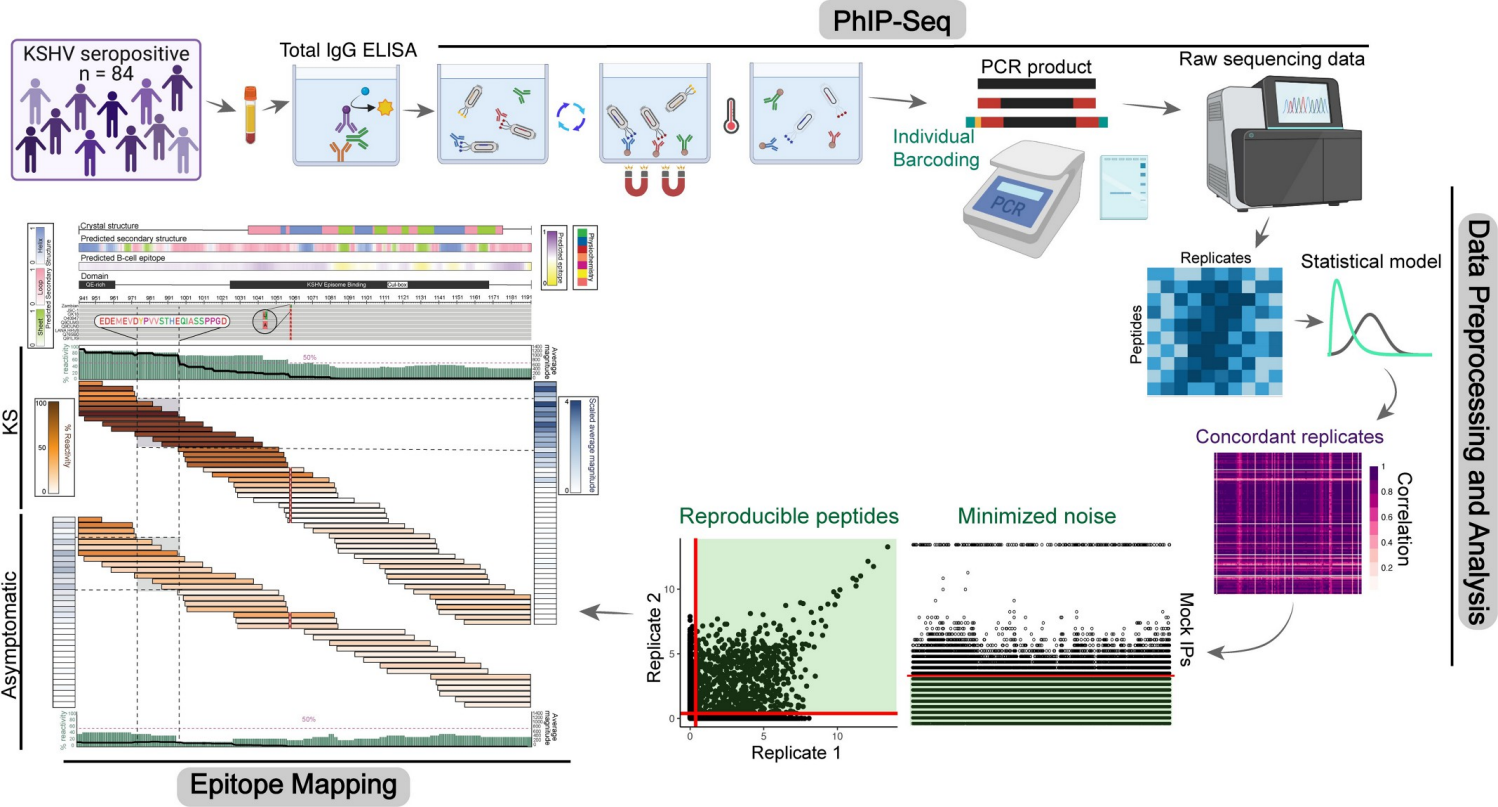
VirScan experiment and data analysis workflow. Phage immunoprecipitation and sequencing (PhIP-Seq) is carried out by first quantifying the levels of total IgG by ELISA, incubating the phage and plasma while rotating to allow complex formation, pulling down the phage-Ab complexes using magnetic beads and washing away the unbound phage, and finally, PCR amplification of the phage DNA followed by sequencing. The sequencing data is aligned to the reference sequences and a peptide by replicate count matrix is generated. The count data was fit to a Gamma-Poisson distribution to obtain residual p-values that represented the enrichment of a peptide above a significance-level cutoff, termed reactive peptides. Peptides were further filtered by removing peptides that had high non-specific binding in mock IPs. Samples were also filtered based on replicate correlations. Finally, statistically significant and reproducible peptides with highly concordant sample replicates were selected for downstream analyses and viral epitope mapping of KSHV-LANA. Created with BioRender.com.

The antibody (Ab) response against KSHV-LANA was mapped using VirScan and samples from sub-Saharan African (SSA) study cohorts in Zambia and Tanzania (**Fig 1**). The cohort included 16 HIV^-^ Kaposi Sarcoma patients (Endemic KS, EnKS) and 46 HIV^+^ Kaposi Sarcoma patients (Epidemic KS, EpKS). The EnKS cohort was significantly older (*Mann-Whitney*, *p = 0*.*0003*) and had a significantly greater proportion of males compared to EpKS cohort (**Table 1**, *Fisher’s Exact Test*, *p = 0*.*012*) reflecting the epidemiology observed in SSA where EpKS is more prominent, while EnKS occurs more frequently in older males [1]. In addition to the KS individuals, 22 KSHV^+^ asymptomatic (KS^-^) individuals were included. To control for both the EnKS and EpKS groups, 11/22 of the asymptomatic individuals were HIV^-^ and 11/22 were HIV^+^. The median age (*Mann-Whitney*, *p>0*.*05*) and proportion of males (*Fisher’s Exact*, *p>0*.*05*) were not significantly different between the KS and asymptomatic groups. Furthermore, all HIV^+^ patients included in this study were on antiretroviral therapy (ART), with self-reported adherence in all but two individuals. The extent of immunosuppression, as reflected in HIV viral load, reduced CD4 counts, or longer duration of HIV infection might have impacted LANA (and other) Ab responses; however, no significant difference in CD4 count, HIV plasma viral load, or duration of HIV infection was evident (**Table 1, S4 Fig,** *Mann-Whitney*, *p>0*.*05*).

**Table 1:** Cohort demographics (N = 84). Significant comparisons are denoted: ^a^EnKS vs. EpKS, ^b^Asymptomatic HIV^-^ vs. HIV^+^, ^c^KS vs. Asymptomatic. P-values are denoted: ****p<0.*0001*, ***p<0.*001*, **p<0.*01*, *p<0.*05*

	Kaposi Sarcoma (KS, n = 62)		Asymptomatic KSHV^+^ (n = 22)	
	HIV^-^ (EnKS, n = 16)	HIV^+^ (EpKS, n = 46)	HIV^-^ (n = 11)	HIV^+^ (n = 11)
	Median (IQR)	Median (IQR)	Median (IQR)	Median (IQR)
Variable	or n (%)	or n (%)	or n (%)	or n (%)
**Demographic**				
**Age (years)** ^a***^	54 (33.75)	32.5 (11)	33 (5)	43 (12)
**Gender** ^a*^				
Female	1 (6%)	19 (41%)	6 (55%)	4 (36%)
Male	15 (94%)	27 (59%)	5 (45%)	7 (64%)
**HIV**				
**Duration (days)**	-	180 (761.3)	-	545 (1615)
**ART Adherence**				
Always	-	42 (92%)	-	11 (100%)
Mostly	-	2 (4%)	-	0 (0%)
Rarely	-	2 (4%)	-	0 (0%)
**CD4 Count**	-	210 (276.5)	-	360 (191.8)
< 200	-	22 (48%)	-	1 (9%)
200–500	-	17 (37%)	-	6 (55%)
> 500	-	6 (13%)	-	1 (9%)
Unknown	-	1 (2%)	-	3 (27%)
**Viral load (cps/mL)**	-	1458 (17532.7)	-	103.5 (43)
Undetectable	-	19 (41%)	-	6 (55%)
Unknown	-	13 (28%)	-	3 (27%)
**Antibody**				
**Total IgG (mg/mL)** ^a*^	6.279 (5.103)	11.044 (8.362)	8.388 (1.63)	8.77 (1.433)
**KSHV Ab Titer** ^c****^	1280 (2240)	1280 (1280)	160 (280)	40 (280)
Low	1 (6%)	9 (20%)	6 (55%)	8 (73%)
Medium	9 (56%)	26 (56%)	5 (45%)	2 (18%)
High	6 (38%)	11 (24%)	0 (0%)	1 (9%)
**Sequencing Data**				
**Reads/Replicate** ^b*^	1,765,070 (385,470)	1,736,288 (452,445)	1,821,327 (243,137)	1,500,691 (635,943)

To normalize total immunoglobulin G (IgG) between the significantly elevated levels in EpKS compared to EnKS subjects (*Mann-Whitney*, *p = 0*.*0144*), a total IgG of 2μg was input into each PhIP-Seq assay. Each precipitation resulted in >850,000 reads/replicate, and the number of reads was not significantly different between KS and asymptomatic groups (**Table 1**, *Mann-Whitney*, *p>0*.*05*). Greater than 95% of reads aligned with the reference sequences suggesting there were no contamination or amplification issues. Additionally, sequencing of 32 mock immunoprecipitations (PBS-only, mock IPs) revealed uniform distribution of the phage library where >99.6% of the expected peptides were represented. From these data, we demonstrated that the phage library population was not bottlenecked during amplification.

There were 224 KSHV-LANA peptides in the VirScan library. Of these 224 peptides, 59 were non-unique amino acid sequences, meaning that Ab precipitation would be non-discriminant between them. Thus, the resulting counts were consolidated with each matching peptide of the 165 remaining for downstream analyses. One of the 165 peptides was missing in all 32 mock-IPs, and thus was likely lost during library amplification. Finally, 9 peptides were filtered out due to high non-specific binding in the mock-IPs (**S1 Fig**) leaving 155 KSHV-LANA peptides for downstream analyses. At least one KS patient reacted to 151/155 potential target peptides, compared to recognition of 130/155 potential peptides in the asymptomatic individuals.

As previously shown, KSHV-specific Ab titer, determined using a BC3 monoclonal-enhanced immunofluorescence assay (mIFA) [7–10], was significantly higher in KS compared to asymptomatic individuals (**Table 1**, *Mann-Whitney*, *p<0*.*0001)*. While the number of reactive peptides per patient, i.e., *breadth*, significantly differed between low, medium, and high total KSHV Ab titer individuals (**S2 Fig**, *Kruskal-Wallis*, *p = 0*.*0006*), titer only moderately correlated with breadth (**S2 Fig**, *Spearman r* = 0.3752, *p = 0*.*0004*) and weakly correlated with log-scaled magnitude (**S2 Fig**, *Spearman r* = 0.1124, *p = 0*.*3087*) indicating that anti-LANA repertoire differences between KS and asymptomatic groups were not solely attributable to titer differentials. Importantly, KSHV Ab titer encompasses the Ab response against all KSHV components, whereas in this study, only anti-LANA responses were quantified. While the breadth and magnitude of response against LANA have an association with KSHV Ab titer, they cannot completely explain the titer, suggesting that other factors (i.e., the response to other KSHV proteins) are also contributing factors to KSHV Ab titer.

### KSHV-LANA can be subdivided into N-terminal, Central Repeat, and C-terminal regions

**Fig 2:**
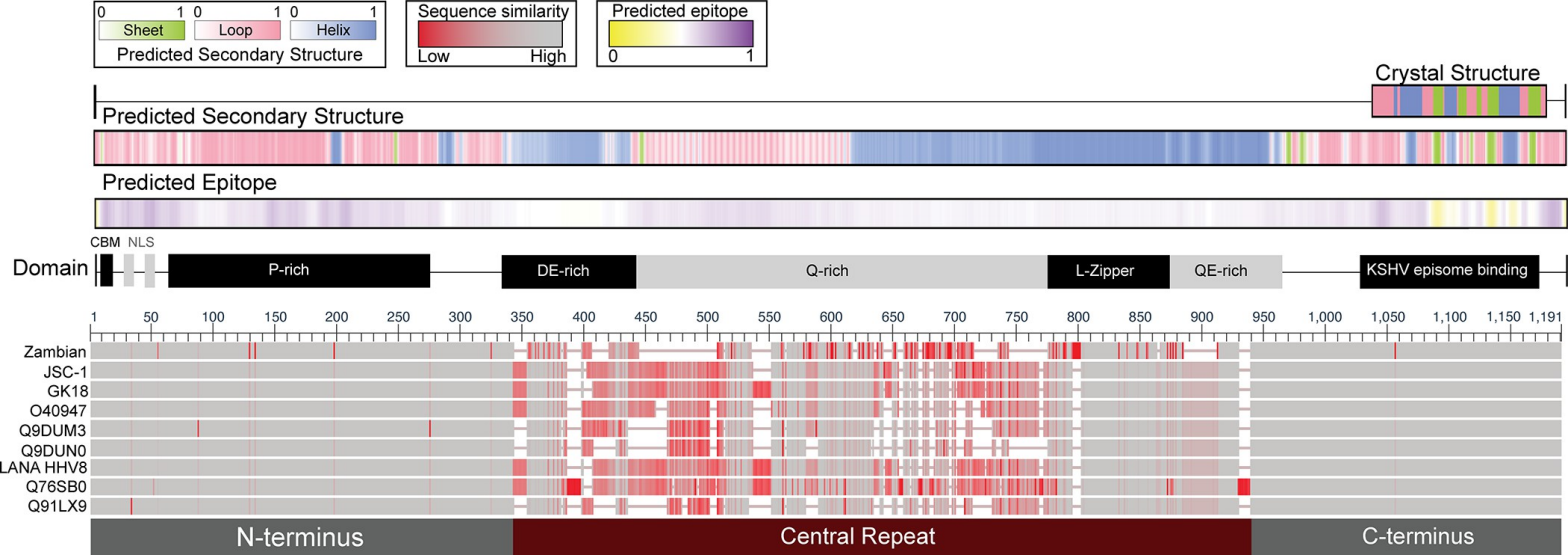
KSHV-LANA protein multiple sequence alignments. The reference LANA sequences (70% Zambian consensus, JSC-1, and GK18) and input library sequences (indicated by their UniProtKB IDs) were aligned, and residues were colored based on their sequence similarity; gray indicates 100% sequence identity, red represents no similarity at a given position. Computationally predicted and crystal secondary structures are annotated and color-coded by type: sheet (green), loop (pink), or helix (blue). Predicted secondary structures are represented in color gradients representing the probabilities (minimum [0; white] and maximum [1; sheet (green), loop (pink), or helix (blue)]). Additionally, the BepiPred-2.0 tool was used to map probabilities of a predicted B-cell epitope. Lastly, functional motifs/domains are annotated directly above the aligned sequences. *CBM*: *Chromosome binding motif*, *NLS*: *Nuclear localization sequence*, *P-rich*: *Proline-rich region*, *DE-rich*: *Aspartic/Glutamic acid-rich region*, *L-Zipper*: *Leucine zipper motif*, *QE-rich*: *Glutamine/Glutamic acid-rich region*.

Since LANA has been partially sequenced many times, the phage library incorporated six different isolates of LANA (UniProtKB accession: O40947, Q9DUM3, Q9DUN0, Q76SB0, Q91LX9, and Q9QR71). When these isolates were compared with common KSHV reference sequences, (GK18 and JSC-1), coverage of the entire LANA protein was evident in the phage library (**Fig 2**). Additionally, LANA is conserved at the N- and C-termini but varied in length and sequence in the central repeat region (**Fig 2**). Due to the nature of the repeat sequences, the central repeat region has been difficult to sequence and align with confidence. This resulted in ambiguous mapping of peptides in this region and hindered analysis of epitopes. For downstream analyses, we divided LANA into three domains based on multiple sequence alignment (MSA) results: N-terminus [MSA 1–342], central repeat region [MSA 343–940], and C-terminus [MSA 941–1191] (**Fig 2**). The Zambian consensus sequence contained six point mutations in the conserved N- and C-termini that were not represented by the isolates in the library, indicating the peptides presented by the phage library were representative of LANA in the virus to which the study cohort would most likely have been infected (see Zambian, **Fig 2**).

The LANA C-terminus contains the KSHV episome binding site and is the only portion of the LANA protein for which a crystal structure has been determined (PDB: 4UZB) [11]. That model shows the LANA C-terminus is mostly random coil, with interspersed helices and beta-sheets. The secondary structure derived from the crystal structure largely agrees with secondary structure predictions, (72% of amino acids accurately predicted) (**Fig 2**). For this reason, the predicted secondary structure of the remaining regions of LANA that have not been crystalized was likewise inferred. The LANA N-terminus contains the chromosome binding site, nuclear localization sequences, and a proline (P)-rich region. The N-terminal region is predicted to be predominantly random coil, especially in the proline-rich region. In contrast, the central repeat region of LANA while harboring regions of random coil, also contains large helical regions, likely resulting from the various repeats and leucine zipper motifs (**Fig 2**). Interestingly, while LANA is known to be a highly antigenic protein [12,13], an advanced B cell epitope prediction algorithm, BepiPred 2.0, did not identify any particular region of LANA to be highly antigenic (see epitope prediction score, **Fig 2**). To demonstrate that VirScan can be used to map B cell epitopes of KSHV, an epitope map of the Ab response against KSHV-LANA was built to investigate which sequences of the protein contribute to its antigenicity and how well such repertoires correspond to predicted B cell epitopes, and to previous empirical Ab-response data.

### The KSHV-LANA central repeat and C-terminal regions show high antigenicity

The 151 validated LANA-derived peptides in the VirScan library were partitioned into N-terminus, C-terminus, and central repeat region resulting in 19/151 peptides in the N-terminus, 48/151 peptides in the C-terminus, and 84/151 peptides in the central repeat region. The Ab response to each region was compared in multiple ways. First, the percentage of patients reactive to at least one of the peptides derived from each of the three regions was calculated and compared between KS and asymptomatic controls: 52% of KS patients and 46% of asymptomatic controls were reactive to at least one peptide within the N-terminus, 69% and 68% in the central repeat region, and 89% and 73% in the C-terminus, respectively. No significant difference in percent patient reactivity between KS patients and asymptomatic controls was observed in any of the regions (*Fisher’s Exact*, *p>0*.*05*), and the C-terminus was the most consistently targeted region among both KS patients and asymptomatic controls (*Chi-square*, *p<0*.*0001*).

**Fig 3:**
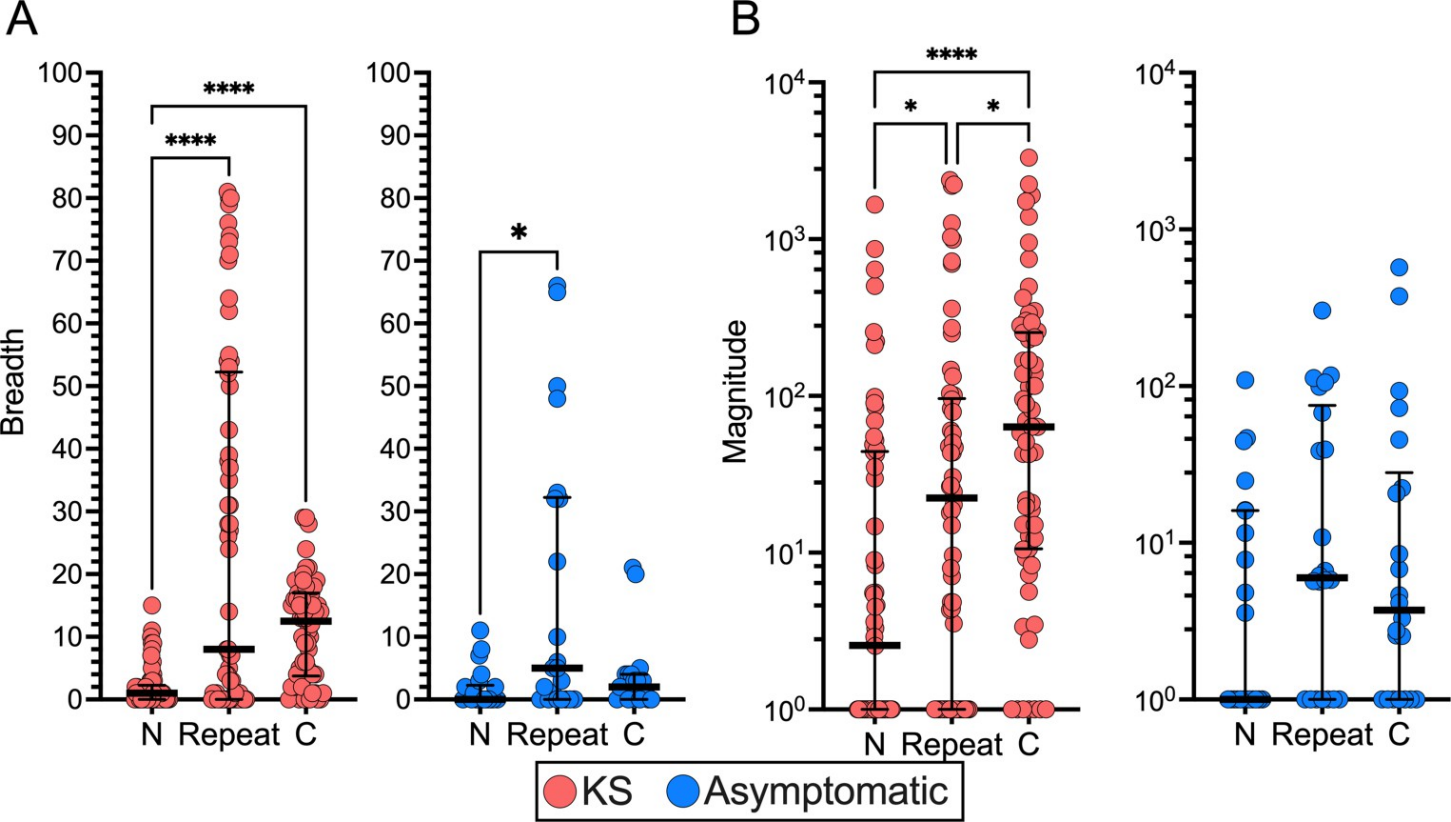
Breadth and magnitude of KSHV-LANA responses across regions. Comparison between KSHV-LANA responses at different regions in KS and asymptomatic individuals based on (A) breadth of response representing the number of reactive peptides, and (B) magnitude of response representing the frequency of which a reactive peptide was targeted (*Kruskal-Wallis post-hoc tests with Dunn’s correction)*. Significance levels are indicated with asterisks, where *p<0*.05 *, *p<0*.01 **, *p<0*.001 ***, *p<0*.0001 ****.

Second, the sum of reactive peptides per sample (*breadth*) for each region was calculated. For KS patients, breadth was significantly higher in the C-terminal and central repeat regions when compared to the N-terminus (**Fig 3A**, *p<0*.*0001*). In asymptomatic controls, only the central repeat region had significantly higher breadth than the N-terminus (**Fig 3A**, *p = 0*.*0185*). The loss of significance was likely due to an overall significantly lower total LANA breadth in asymptomatic controls compared to KS patients (**S3 Fig**, *p = 0*.*0452*). Furthermore, the breadth was moderately able to linearly separate KS and asymptomatic by principal component analysis (PCA), explaining ~43% of the total variance (**S3 Fig**). Lastly, the average magnitude of reactive peptides per sample for each region was calculated. The average magnitude represents how many times each reactive peptide was targeted by an Ab. Similar to breadth, the magnitude was significantly different across all three regions in KS patients, where the C-terminus was the most frequently targeted region (**Fig 3B**). On the contrary, there was no significant difference between the three regions of LANA in asymptomatic controls (**Fig 3B**). This data demonstrates that the central repeat region of LANA was nearly as antigenic as the C-terminus. However, due to the lack of confidence in amino acid position assignments, responses to the central repeat region could not be confidently mapped (**Fig 2**) [12]. Notably, no single peptide from the repeat region was consistently hit across the cohort and no shared converging repeat pattern was observed amongst the top reactive peptides. Nevertheless, epitope mapping of the N- and C-terminal domains readily identified the peptides and amino acids responsible for LANA’s antigenicity.

### The KSHV-LANA N-terminus exhibits weak antigenicity

**Fig 4:**
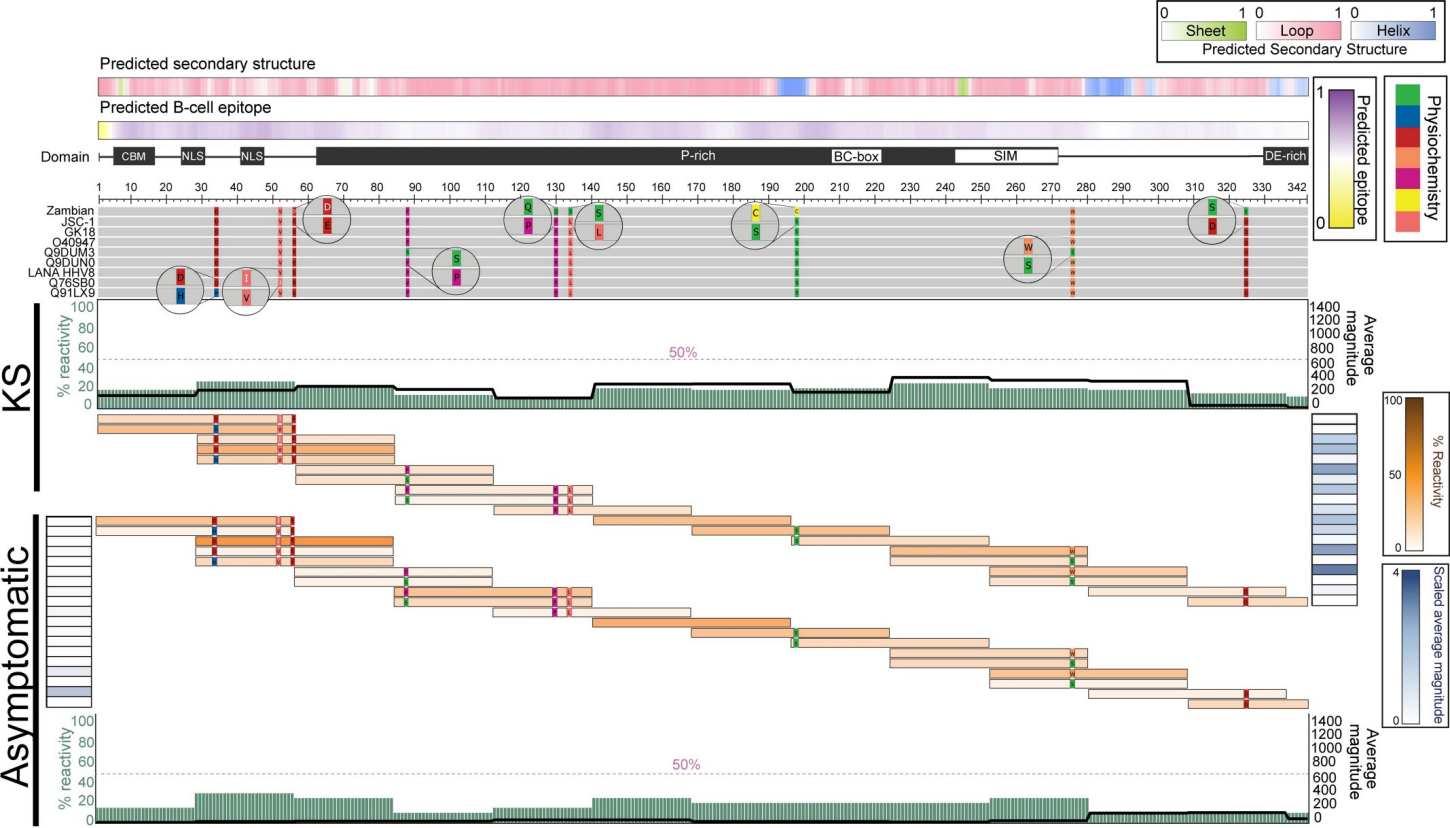
KSHV-LANA N-terminus epitope mapping in KS and asymptomatic individuals. The multiple sequence alignment (MSA) of reference sequences followed by the input library sequences is shown where gray represents conservation among the sequences. Mismatched residues are colored by their physiochemical properties as defined by the Zappo color scheme (green: hydrophilic, salmon: aliphatic/aromatic, orange: aromatic, fuchsia: conformationally special, yellow: cysteine only, red: negatively charged, blue: positively charged). Predicted secondary structures are represented in color gradients representing the probabilities (minimum [0; white] and maximum [1; sheet (green), loop (pink), or helix (blue)]). BepiPred-2.0 scores representing predicted B-cell epitope probabilities, along with functional motifs/domains are annotated directly above the aligned sequences. In addition, each peptide that was targeted by at least one individual was mapped and colored based on the percent cohort reactivity (brown gradient). Adjacent to the peptide responses of each group, scaled average magnitudes are shown in blue gradient. Moreover, percent patient reactivity and average magnitude of responses are calculated at the amino-acid level, represented by the sage bars and black lines in each group, respectively. *CBM*: *Chromosome binding motif*, *NLS*: *Nuclear localization sequence*, *P-rich*: *Proline-rich region*, *BC-box*: *associated to Elongin B/C motif*, *SIM*: *Small ubiquitin-related modifier (SUMO) Interacting motif*, *DE-rich*: *Aspartic/Glutamic acid-rich region*.

The LANA N-terminus had lower overall breadth and magnitude when compared to the C-terminus and central repeat regions (**Fig 3**), suggesting it was the least antigenic region of the LANA protein. To determine whether Ab recognition of the N-terminus was uniformly low or if there was a focal point of antigenicity, the peptides contained in the N-terminal domain were mapped to the reference sequences and the percent patient reactivity and average magnitude were overlayed (**Fig 4**). The maxima of patients reactive to each peptide in the N-terminus never reached 25%, demonstrating that there was no epitope consistently targeted in either the KS or asymptomatic groups. To confirm this was not an effect of having overlapping peptides, the percent patient reactivity per residue was calculated. For each amino acid residue, if a patient was reactive to at least one peptide encompassing that residue, they were considered reactive. The same logic was applied to the magnitude, such that, for all patients considered reactive to a residue, the magnitudes of the individual’s responses to each of the peptides encompassing that residue were averaged. The per residue percent patient reactivity increased slightly, but remained <30%, confirming that there were no consistently targeted epitopes in the LANA N-terminus (**Fig 4**). Similarly, the average magnitude was low across the entire N-terminus at both the peptide and amino acid levels (**Fig 4**). Regions with the highest percent patient reactivity in the N-terminus were the NLS and a portion of the Proline-rich region in both KS and asymptomatic (**Fig 4**). Broad, low magnitude to the N-terminus of LANA was detected with a slight increase in KS, suggesting the N-terminal region of the LANA protein is not stimulating an efficient Ab response in either group. Additionally, peptides containing the five point mutations in the N-terminus showed similar percent reactivities, indicating that they did not skew the antigenicity profiles.

### The first half of the KSHV-LANA C-terminus contains a consistently targeted epitope

**Fig 5:**
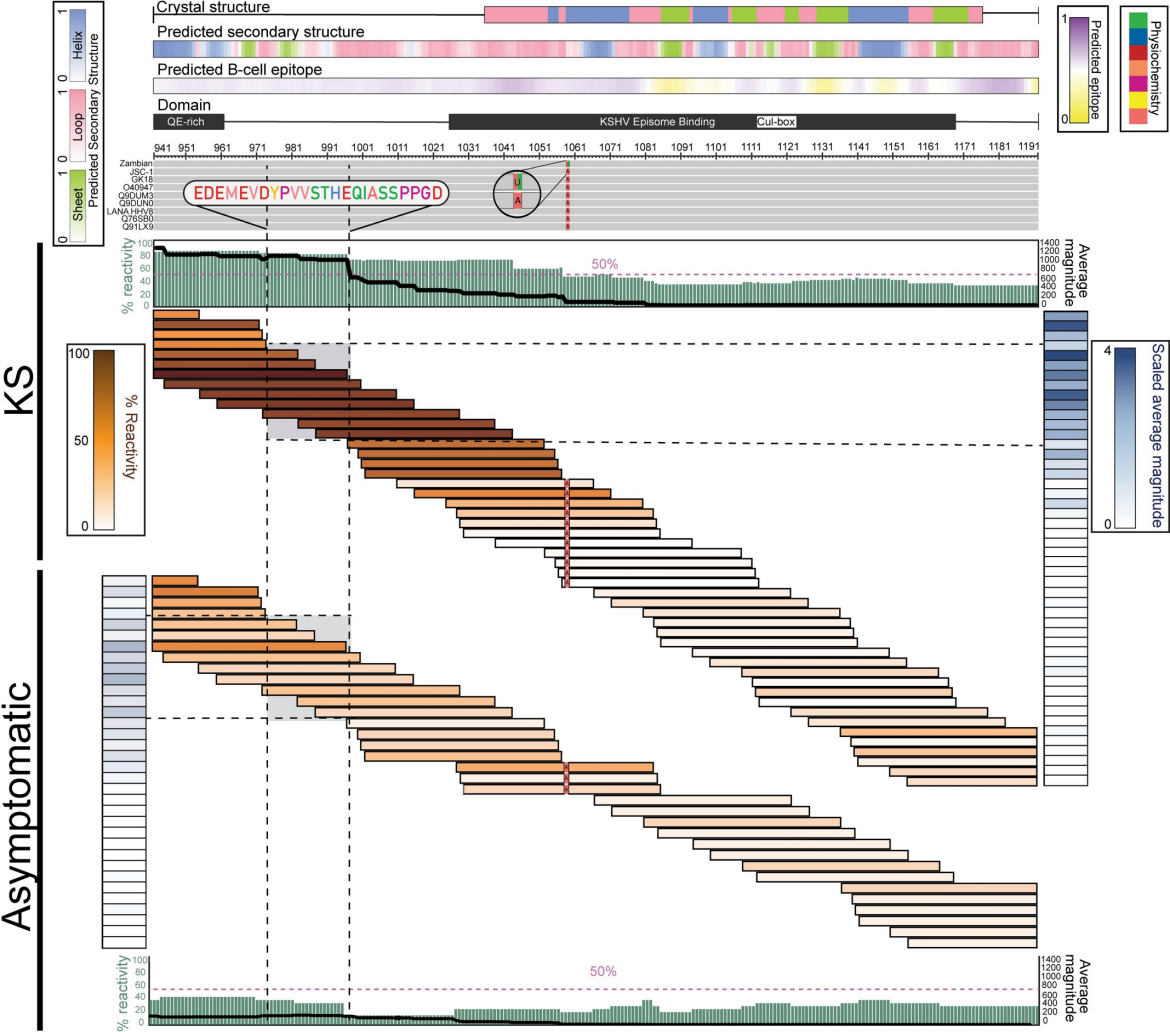
KSHV-LANA C-terminus epitope mapping in KS and asymptomatic individuals. The multiple sequence alignment (MSA) of reference sequences followed by the input library sequences is shown where gray represents conservation among the sequences. Mismatched residues are colored by their physiochemical properties as defined by the Zappo color scheme (green: hydrophilic, salmon: aliphatic/aromatic, orange: aromatic, fuchsia: conformationally special, yellow: cysteine only, red: negatively charged, blue: positively charged). Predicted secondary structures are represented in color gradients representing the probabilities (minimum [0; white] and maximum [1; sheet (green), loop (pink), or helix (blue)]). BepiPred-2.0 scores representing predicted B-cell epitope probabilities, along with functional motifs/domains are annotated directly above the aligned sequences. In addition, each peptide that was targeted by at least one individual was mapped and colored based on the percent cohort reactivity (brown gradient). Adjacent to the peptide responses of each group, scaled average magnitudes are shown in blue gradient. Moreover, percent patient reactivity and average magnitude of responses are calculated at the amino-acid level, represented by the sage bars and black lines in each group, respectively. LANA_974-997_ is highlighted and the amino acid sequence is shown. *QE-rich*: *Glutamine/Glutamic acid-rich region*, *Cul-box*: *associated with Cullin-5*.

The overall Ab-response to the C-terminus of LANA was higher than the response to the N-terminus (**Fig 3**), and like the N-terminus, the C-terminal peptides were mapped to the reference sequences to ascertain if the C-terminal reactivity was focal or broad. This mapping revealed that the first half of the C-terminus was highly targeted in KS patients (**Fig 5**). While the same recognition pattern was detected in the asymptomatic controls, it was less consistent and occurred at lower magnitude (**Fig 5**). More specifically, the nine overlapping peptides spanning LANA_974-997_ [EDEMEVDYPVVSTHEQIASSPPGD] were the most consistently targeted and had high average magnitudes across KS patients. LANA_974-997_ was also highly recognized in asymptomatic controls, but in reactivity was similar to LANA_941-973_ [QELEEVEEQEQQGVEQQEQETVEEPIILHGSSS]. While neither region was predicted to be highly antigenic by the BepiPred 2.0 tool, our data demonstrate these are the most antigenic LANA epitopes in KSHV infected individuals (see Predicted B cell epitope, **Fig 5**). B cell epitope prediction at this region was only marginally positive since the BepiPred2.0 tool associates confident random coil structure to higher likelihood of a potential epitope, and the structural predictions included both helices and beta-strands in addition to random coil. The predicted secondary structure of LANA_974-997_ was mostly random coil with a beta-strand near the beginning, while LANA_941-973_ was mostly helical with a beta-strand towards the end. To conclude, the Ab repertoire against the LANA C-terminus is against a pair of specific epitopes rather than the low, broad response to the N-terminus (**Fig 5**). The focused response against the C-terminus is likely targeting the highly antigenic epitope within LANA_941-997,_ a response that may be refined or reinforced in KS.

### The effect of HIV co-infection on the anti-LANA response

**Fig 6:**
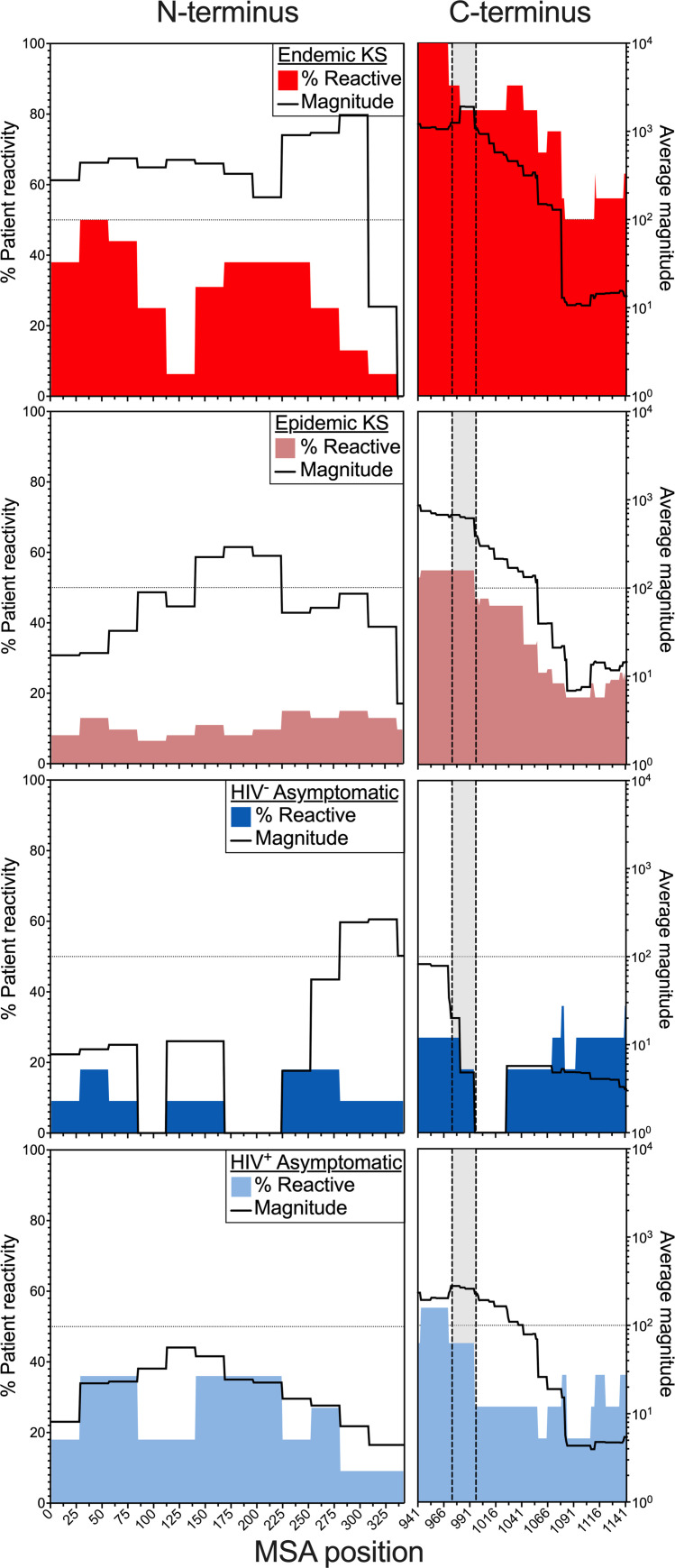
Comparison of percent patient reactivity and the average magnitude of Ab response per residue by KS and HIV status. The colored areas represent the percent of patients that were reactive to at least one peptide containing that residue, while the black lines represent the log-scaled average magnitude [log(–log(*p*))] at that residue for EpKS, EnKS, HIV^+^ asymptomatic, and HIV^-^ asymptomatic individuals in both the N- and C-termini. The vertical bars highlighted within the dotted lines span the epitope, LANA_974-997._

To investigate the effect of HIV infection on the Ab response, we stratified the KS and asymptomatic groups by HIV status and comparatively evaluated the percent patient reactivity and average magnitude at the residue level (**Fig 6**); where a high percent reactivity indicates the given region was consistently targeted by patients across the cohort, and a high average magnitude indicates the given region was highly targeted within the patients. Notably, all four groups followed a similar pattern of reactivity across both the N- and C-termini with varying strength and consistency (**Fig 6**). While the overall N-terminal breadth and magnitude were not significantly different between EnKS and EpKS (**S3 Fig**), a more focused response was evident in the EnKS patients as indicated by peaks in percent patient reactivity and average magnitude (**Fig 6**). In contrast, EpKS patients followed a similar pattern but showed less consistency with lower, more uniform magnitude (**Fig 6**). This suggests that in the absence of HIV infection and recognized immune suppression, EnKS patients were able to develop a more specific, focused response that would not have been evident without the epitope mapping. Moreover, the HIV^+^ asymptomatic individuals followed the pattern of EnKS patients and had a more focal response in the N-terminus, while the HIV^-^ asymptomatic controls had scattered patient-specific reactivity across the N-terminus (**Fig 6**). However, there was no significant differences in either breadth or magnitude between the HIV^-^ and HIV^+^ asymptomatic individuals (**S3 Fig**) suggesting that HIV infection had a subtle effect in the absence of KS.

Importantly, all four groups had more consistent responses to the C-terminus compared to the N-terminus (**Fig 6**). EnKS patients had the highest reactivity across the C-terminus as denoted by a very consistent response (**Fig 6**) and a significantly greater breadth (**S3 Fig**). The magnitude of responses was also significantly elevated in the EnKS patients compared to HIV^-^ asymptomatic controls, suggesting that in the absence of HIV infection, the anti-LANA C-terminal response is elevated in disease (**S3 Fig**). Furthermore, EnKS patients responded more consistently to the first half of the C-terminus than the second half, as did EpKS patients and HIV^+^ asymptomatic individuals (**Fig 6**), while the HIV^-^ asymptomatic controls had an inconsistent, low response. The highly antigenic public epitope LANA_974-997_ identified in the KS and asymptomatic groups (**Fig 5**) had the best combination of high percent patient reactivity and average magnitude across the four groups, but LANA_941-973_ should also be noted as a region of high responsiveness (**Fig 6**).

In summary, the overall pattern of responsiveness was similar in EpKS, EnKS, HIV^+^ asymptomatic, and HIV^-^ asymptomatic individuals (**Fig 6**). The first half of the C-terminus (LANA_941-997_) was the most likely target of LANA antibodies in patient plasma regardless of HIV and KS status (**Figs 5 and 6**). Nonetheless, EnKS patients showed the highest reactivity, and the HIV^-^ asymptomatic controls showed the lowest reactivity across both termini (**Fig 6**). Thus, HIV infection in the context of KS appeared to hamper the Ab response, while HIV^+^ asymptomatic individuals had stronger, more consistent anti-LANA responses that better mirrored those detected in KS patients compared to HIV^-^ asymptomatic controls. Overall, these high-throughput phage display data demonstrate that HIV co-infection and disease (KS) do have effects on the overall anti-LANA response and highlight the value of epitope mapping to elucidate the breadth and depth of the complete KSHV Ab repertoire in infection and neoplastic disease.

## Discussion

Using a novel and high-throughput phage-display technology, VirScan, we generated the first high-resolution epitope map of the KSHV-LANA protein using cohorts representing KS and asymptomatic KSHV infection, each with and without HIV co-infection. VirScan supported fine resolution epitope mapping of the N- and C-terminal domains of LANA, which recapitulates previous work, but yields higher resolution mapping that demonstrates the utility of this phage display technique to identify antigenic epitopes with therapeutic potential in other KSHV proteins. The beginning of the LANA C-terminus immediately following the central repeat region, LANA_941-997_, was consistently recognized in both KS and asymptomatic KSHV-infected individuals, with or without HIV co-infection, suggesting it is the predominant epitope responsible for LANA’s antigenicity. Although KS patients had higher reactivity compared to asymptomatic individuals, the two cohorts had a similar pattern of reactivity across the LANA protein. Importantly, while it has been previously shown that KS patients have higher KSHV-specific Ab titers [7–10], we demonstrated that total KSHV Ab titer had a weak-to-moderate association with the breadth or magnitude of the anti-LANA response, respectively.

Our findings support several prior studies that utilized recombinant proteins to characterize the overall reactivity to LANA and the antigenicity of its three constituent domains. For example, Labo, et al. systematically expressed and purified 72 KSHV ORFs in recombinant systems and found LANA (ORF73) to be the most consistently targeted KSHV protein [2]. Similarly, Zheng, et al. used a protein microarray to compare the Epstein-Barr Virus (EBV) and KSHV humoral responses among healthy controls, EpKS patients, and lymphoma patients [12]. Due to the inability to express the central repeat region in yeast, the N- and C-termini of LANA were expressed separately on the microarray slide, enabling comparisons of reactivity between the two regions. The C-terminal region of LANA was not only highly antigenic, but it was recognized considerably more frequently than the N-terminus, as in our study [12]. Lastly, in an effort to design a sensitive assay to screen KSHV-infected individuals, Olsen, et al. measured the reactivity to 17-mer biotinylated peptides derived from LANA using ELISA [13]. Although their assays did not reach the desired sensitivity, they observed higher reactivity against the LANA central repeat and C-terminal regions compared to the N-terminus.

Although anti-LANA breadth indicated that the central repeat region was highly antigenic, individual peptide sequences were not consistently targeted and the peptides from this region could not be confidently mapped due to the inability to discriminate recognition of one iteration of a repeat from another. For example, the most consistently targeted peptide within the repeat region was only recognized by 56% of KS patients, whereas the most consistently targeted peptide containing LANA_974-997_ was recognized by 74%. Interestingly, LANA_974-997_ is unique to KSHV LANA, sharing no sequence similarity with proteins from other viral or nonviral species. Since epitopes primarily reside on the surface of proteins, they are found often in random coils which promote exposure [14], and numerous studies on the sequence composition of B cell epitopes have demonstrated that epitopes are enriched in regions composed of polar and charged amino acids [15]. Consistent with these concepts, LANA_974-997_ predominantly consists of polar and charged amino acids and is predicted to adopt random coil structure (~63%). Moreover, LANA_941-997_, was consistently recognized in both KS patients and asymptomatic controls, regardless of HIV co-infection. High-resolution epitope mapping revealed a subtle increase in anti-LANA reactivity in HIV co-infected asymptomatic individuals compared to those that were infected with KSHV alone, an aspect of the response profiles that was otherwise indistinguishable. This increase could result from immune activation and/or increased KSHV lytic reactivation in HIV^+^ individuals. An opposite trend was observed in KS, where the HIV^-^ EnKS patients exhibited higher anti-LANA responses. The lower response to LANA_941-997_ in EpKS patients may be explained by the progression of HIV infection to an HIV-associated malignancy prior to initiation of ART or the continued disease progression and immune suppression in untreated subjects within the cohort. Of note, prior immunosuppression is more likely to affect the Ab response, and as this study used a retrospective cohort, we did not have access to the participants’ nadir CD4 counts. However, there was a weak-to-moderate correlation of breadth and magnitude with CD4 count (n = 53) measured at the time of sample collection, suggesting that reconstituted CD4 cells support a stronger anti-KSHV Ab response (**S4 Fig**). In a small subset of samples with HIV viral load available and detectable (n = 16), we observed no substantial association of breadth and magnitude of responses with HIV viral load (**S4 Fig**).

Phage display has long been used to map epitopes of proteins, but most often, short peptides (<20 amino acids) that lack potential to form secondary structure are expressed. The high-throughput phage display library used in this study has partially addressed this drawback by expressing 56-mer peptides with 28 amino acid overlap to tile the entire LANA protein. The ideal peptide loaded on a BCR is shorter than the 56-mer peptides used in this study, however, longer peptides enable autonomous folding and adoption of pseudo-conformational epitopes. Thus, the peptides in this study are likely to have adopted more native conformations, but since only continuous epitopes were displayed, epitopes requiring tertiary or quaternary structure would have been missed in our analysis. Additionally, as in other phage display approaches, the linear T7-phage expressed peptides were amplified in bacterial cells, thus it is likely they will lack appropriate post-translational modifications. Therefore, our study is limited to analyzing linear epitopes that lack post-translational modifications. Nonetheless, there are several examples where linear epitopes can be effectively used to induce protective humoral Ab responses [16], and our findings clearly recapitulate those of previous LANA antigenicity studies.

In summary, a high-throughput phage library expressing systematically derived peptides from KSHV LANA was successfully used to generate a fine resolution epitope map and elucidate the epitopes driving LANA’s high antigenicity. Further studies are warranted for investigating why LANA_941-997_ is highly antigenic and if it acts as a potential decoy mechanism or has any therapeutic or diagnostic potential. Additionally, we intend to apply this technique to characterize the linear epitopes in the remainder of the KSHV proteome which will have broader implications, such as the identification of epitopes that can potentially be used to induce protective humoral immune responses.

## Materials and methods

### Ethics statement

The Institutional Review Boards of the University of Nebraska, the Louisiana State University Health Sciences Center-New Orleans, the University of Zambia Biomedical Research Ethics Committee, Tanzania National Institute for Medical Research, and the Ocean Road Cancer Institute approved the study. Written informed consent was obtained from all participants.

### Sample collection

KS patients and asymptomatic KSHV-infected individuals were recruited from Zambia and Tanzania, as described previously [17–20]. Briefly, informed consent was obtained, whole blood samples were collected in EDTA tubes (BD), and plasma was isolated by centrifugation (545*xg* for 15 minutes).

### KSHV serology

To determine KSHV serostatus and titer of the anti-KSHV Ab, we performed a monoclonal-enhanced immunofluorescence assay (mIFA) using stimulated BC3 cells, as previously described [7–10]. Briefly, BC3 cells were fixed with 4% paraformaldehyde, permeabilized with 0.1% Triton X-100, and spotted on Teflon-coated 12-well slides. Plasma was heat-inactivated at 56°C for 30 minutes and diluted at 1:40 in phosphate-buffered saline (PBS) for the determination of serostatus. If the plasma sample was positive at 1:40 dilution, further two-fold dilutions were carried out to ascertain the end-point titer. The appropriate dilution of plasma was added to the slide and incubated at 37°C for 30 minutes, followed by the addition of mouse anti-human IgG (CRL 1786, American Type Culture Collection) and Cy2-conjugated donkey anti-mouse IgG. Additionally, Evans blue was used as a reference stain to visualize cells. Plasma was considered positive if two of three independent readers called the sample positive.

### HIV viral load

HIV-1 plasma viral load was measured as previously described [21,22]. Briefly, the QIAamp viral RNA mini kit with on-column DNase I treatment (Qiagen, cat #52904) was used to extract viral RNA from plasma. HIV-1 LTR was quantified using qPCR and AcroMetrix HIV-1 High Control samples for standards (Thermo Scientific, cat #964001). HIV-1 copies/mL was calculated based on the standard curve in QuantStudio Design and Analysis Software (ThermoFisher). Statistical analyses were performed in GraphPad Prism v9.3.1.

### Phage Immunoprecipitation and Sequencing (PhIP-Seq)

The T7-Vir3 phage library was kindly provided to us by Dr. Stephen J. Elledge at Harvard Medical School. The library has been previously described [5]. Once the library was received, it was amplified and titered as directed by the manufacturer using BLT5403 (Novagen T7Select System) [5,6,23]. Total IgG was quantified using ELISA (Invitrogen, #BMS2091) according to the manufacturer’s instructions. PhIP-Seq protocol was performed as previously described [5,6] and consists of three major steps: complex formation, immunoprecipitation, and library DNA preparation. Eight mock precipitations (mock IPs; PBS) were run on each plate, and each sample was run in duplicate to ensure reproducibility. First, the plasma samples were diluted to 0.5μg/μL total IgG and 96-well deep well plates were blocked with 3% BSA in TBST (Tris-buffered saline, 0.1% Tween-20). The phage library was thawed at 4°C and diluted to 2x10^10^ pfu/mL, such that 10^5^ pfu per library member would be present for each 1mL reaction. The library was distributed to each well of the blocked deep well plate, and 2μg of IgG (4μL of 0.5μg/μL dilution) from each plasma sample was added to the appropriate well. This mixture of phage library and plasma was incubated rocking end-over-end at 4°C for 20 hours. Next, 40μL of a 1:1 mixture of Protein A and Protein G Dynabeads (Invitrogen, 10008D/9D) was added to each well. The mixture of phage library, plasma, and magnetic beads was incubated at 4°C for 4 hours rocking end-over-end. Then, we placed the plate on a magnetic separation rack (NEB, S1511S) and waited for 2 minutes. After the beads were sequestered, we removed the supernatant consisting of unbound phage and added 400μL of PhIP-Seq Wash Buffer (150 mM NaCl, 50 mM Tris-HCl, and 0.1% (vol/vol) Triton X-100, pH 7.5). This was repeated for a total of three washes, and after the second wash, the bead mixture was transferred to a fresh blocked 96-well deep well plate. After the third wash, the bead mixture was resuspended in 40μL of nuclease-free water and transferred to a 200μL PCR plate. The bead mixture was then heated to 95°C for 10 minutes in a thermocycler, and then stored at -20°C until the next step.

The DNA was prepared for sequencing using three rounds of PCR. The first round performed 30 cycles of amplification, and the second round utilized eight cycles of amplification to add the Illumina adaptors and barcode each well of the 96-well plate. After the second round of PCR, quantitative PCR was performed to ensure that each well had a similar amount of DNA. Once it was determined that the DNA amount was consistent across wells, the barcoded samples were pooled into a single tube and subjected to a third round of PCR consisting of a single round of amplification using replenished primers to ensure the library was of uniform length. The third round PCR product was gel purified using the E.Z.N.A. Gel Extraction Kit (Omega Bio-tek, D2500-02). Finally, the gel-purified DNA was sent to the UNMC Genomics core for quality check and sequencing.

The final pool of gel-purified DNA was quantified using Qubit DS DNA HS Assay reagents and a Qubit Fluorometer (Life Technologies), and the size was confirmed using an Agilent 2100 Bioanalyzer. Each of the gel-purified, barcoded DNA pools was the correct amplicon size (≈376bp) and of sufficient DNA concentration. The custom Read1 and Index1 primers were diluted to 0.3μM with sequencing buffer (following the Illumina custom primer guide) and added to the reagent cartridge. The DNA pool was denatured with 0.2N NaOH and adjusted to a final concentration of 1.4pM. The clustering and sequencing were performed on an Illumina NextSeq550 using the 50-cycle, single-end protocol (Mid-output flow-cell). The run was monitored by Illumina Sequence Analysis Viewer and the final FASTQ files were generated after de-multiplexing.

### Data processing and analysis

#### Phage library annotation

The database for the phage library includes annotations for each peptide. These include, but are not limited to, the amino acid sequence, taxonomic identification from strain to kingdom level, protein names, as well as their functional descriptions. Each peptide is assigned a unique ID based on the oligonucleotide sequences, such that entries with identical amino acids can be identified. Duplicate rows, and peptides corresponding to obsolete, redundant, or deleted UniPROT IDs were either removed from the database, or their accession IDs were updated based on the UniProtKB, where possible. Peptides with duplicate amino acid sequences were apparent in the phage library. This was an artifact of including multiple isolates of the same protein where the sequence was identical for a subset of peptides. Thus, another round of curation was applied to the peptides with duplicate amino acid sequences since Abs would not have been able to distinguish between them. The raw read counts for these duplicate peptides were summed, ensuring that the Ab responses quantified represented unique phage-Ab interactions.

### Analysis of PhIP-Seq data

A reference fasta file that only contained unique oligonucleotides was built, and a bowtie index was generated in preparation for aligning the sequencing reads to the library reference sequences. The PhIP-stat tool (https://github.com/lasersonlab/phip-stat) was used to preprocess the raw PhIP-seq data. First, the raw counts for each phage were normalized. A generalized linear model using the Gamma-Poisson distribution was applied to obtain the–*log*_*10*_*(residual p-value)*, MLXP, representing the frequency with which a peptide is targeted. Thus, the MLXP is the magnitude of a phage-Ab response and will be referred to as such throughout this study. Pearson correlation coefficients (*ρ*) between each subject’s paired plasma replicates were calculated using the raw count data, and samples with *ρ*<0.7 were excluded from downstream analyses.

A given peptide was considered reactive if and only if its magnitude was >1.3 (i.e., *p*<0.05) in both replicates. A presence-absence matrix was generated, where a value of one indicated the plasma had an Ab that reacted to the given peptide, and a value of zero indicated a lack of response to the given peptide. The sum of reactive peptides per sample represents the breadth of response. This dichotomization of the data provides a broad overview of patient reactivity against LANA. Subsequently, for each peptide, the magnitude was averaged across the replicates, representing an individual’s magnitude of response against the peptide. Peptides that were non-reactive across all individuals or mock IPs were filtered out. Furthermore, low-quality peptides (i.e., peptides that showed reactivity in >25% of the mock IPs) were filtered out to eliminate spurious non-specific binding events. All statistical analyses were performed in GraphPad Prism v.9.3.1, and additional downstream analyses were performed using the R programming language. All relevant data is included in the supplementary information (S1 Dataset).

### Epitope mapping

Full-length protein sequences of KSHV-LANA variants presented in the library were obtained from the Protein Knowledgebase (UniProtKB). The reference sequences used were the Human herpesvirus 8 strain GK18 (GenBank Accession with version number: AF148805.2; NCBI Reference Sequence: NC_009333.1) [24,25], Human herpesvirus 8 strain JSC-1 clone BAC16 (GenBank Accession with version number: GQ994935.1) [26]. MView was used to generate a 70% sequence identity consensus of the LANA sequences of Zambian patient-derived KSHV [27,28].

The COBALT multiple sequence alignment (MSA) tool was used to generate LANA reference and library MSAs, which highlight the conserved N- and C- termini, and the central repeats. The N- and C-terminal regions were mapped for corresponding reactive peptides using their relative alignment loci. Due to internal repeat region variability across different LANA isolates, the relative start and end locations of the C-terminal region were calculated based on the MSAs. A relative start (or end) site of a reactive peptide was defined by subtracting the start (or end) location of the MSA for the specific LANA sequence from the start location of the peptide itself. After sorting by their relative start sites, the peptides were realigned using COBALT, employing high gap introduction and extension penalties, but without query clusters under advanced parameter options. The outputs were exported in scalable vector graphics (SVG) format for downstream annotation and data visualization.

On both the peptide and amino acid levels, percent patient reactivity and average magnitude of Ab responses were measured. The peptides mapped to the MSAs were displayed as a heatmap to visualize the range of percent patient reactivity against each peptide. For each peptide against which an Ab response was evident, the average magnitude of the Ab response among all responsive patients was calculated. Single amino acid polymorphisms were highlighted in the MSA based on color designations representing the physiochemical properties of the amino acid (Zappo color scheme–green: hydrophilic, salmon: aliphatic/aromatic, orange: aromatic, fuchsia: conformationally special, yellow: cysteine only, red: negatively charged, blue: positively charged–JalView [29]). Where available, the secondary structure defined by previously characterized crystal structure was annotated on the alignment (PDB: 4UZB) [11], along with the predicted secondary structures derived from PredictProtein–Secondary Structure feature (RePROF) [30,31].

Functional motifs and empirically determined domains of LANA were annotated. These include the Histone 2A/B binding domain (the chromosome binding motif), nuclear localization signals (NLS) [32–34], the SUMO-interacting motif (SIM) [33,35], the Elongin B and C box (BC-box) [33,36], and the proline- and the aspartic-glutamic acid-rich regions within the N-terminal region, along with the glutamine-glutamic acid-rich region and the Cullin 5 box (Cul-Box) within the DNA binding domain (DBD) on the C-terminus [33,36]. Finally, results from a B-cell epitope predictor were included using default parameters (BepiPred-2.0) [14], where the epitope prediction scores are represented in probabilities mapped on the alignments as a heatmap.

## Supporting information

S1 Dataset The relevant data used for analysis throughout this study. The sample metadata, LANA-peptide reactivities, and the corresponding magnitude of each reactive peptide are included. Reactive peptides are denoted by “1” and non-reactive peptides by “0”. The sum of reactive peptides per sample represents the breadth of response. The magnitude of response (i.e., *-log(p)* obtained by the Gamma-Poisson model) represents the frequency with which a reactive peptide was targeted. The UniProtKB accession, amino acid sequence, and LANA protein region are provided for each peptide.
(XLSX)

S1 Fig Representation of KSHV-LANA peptides in the input VirScan library. Each plot represents all the peptides from a UniProt entry, and each bar represents the log-scaled counts of a given peptide present in the library from the corresponding UniProt entry. The green bars represent the peptides filtered out due to significant binding in the mock-IPs. The missing peptides (no label on the x-axis) were non-unique amino acid sequences and were thus merged with the peptide that had the matching amino acid sequence.
(TIF)

S2 Fig The relationship between KSHV Ab titer and KSHV-LANA breadth and magnitude. The KSHV Ab titer was determined using mIFA and categorized: low (1:40, 1:80, 1:160), medium (1:320, 1:640, 1:1280), and high (1:2560, 1:5120, 1:10240). (A) LANA Breadth, the number of reactive KSHV-LANA peptides per patient, was compared between titer levels (*Kruskal-Wallis*) and correlated with titer (*Spearman*). (B) LANA magnitude, the average magnitude (MLXP) of the reactive KSHV-LANA peptides per patient, was compared between titer levels (*Kruskal-Wallis*) and correlated with titer (*Spearman*). The 95% confidence interval of the linear regression is shown. Significance levels are indicated with asterisks, where *p<0*.05 *, *p<0*.01 **, *p<0*.001 ***, *p<0*.0001 ****.
(TIF)

S3 Fig KSHV-LANA breadth and magnitude compared between EnKS, EpKS, and HIV^-^/HIV^+^ asymptomatic individuals. The number of reactive peptides and their magnitudes per individual was calculated and compared across disease states, in each group, and across each region within LANA, respectively. Comparison of (A) KSHV-LANA breadth and (B) magnitude across KS and asymptomatic individuals *(Mann-Whitney)* and 2D visualization of the PCA plot. Comparison of (C) KSHV-LANA breadth and (D) magnitude across EnKS, EpKS, KSHV^+^/HIV^-^ and KSHV^+^/HIV^+^ individuals within each region *(Kruskal-Wallis)*. Significance levels are indicated with asterisks, where *p<0*.05 *, *p<0*.01 **, *p<0*.001 ***, *p<0*.0001 ****.
(TIF)

S4 Fig Associations of KSHV-LANA breadth and magnitude with HIV plasma viral load and CD4 counts in HIV^+^ KS/Asymptomatic individuals. Both KSHV breadth and magnitude of Ab responses were tested for association with (A) CD4 count (n = 53) and (B) HIV viral load (n = 41).
(TIF)
