# Supplementary figures and images for: Antibody epitope profiling of the KSHV LANA protein using VirScan
Source: PLoS Pathog. 2022 Dec 19;18(12):e1011033. doi: 10.1371/journal.ppat.1011033 (PMC9810164; doi:10.1371/journal.ppat.1011033)

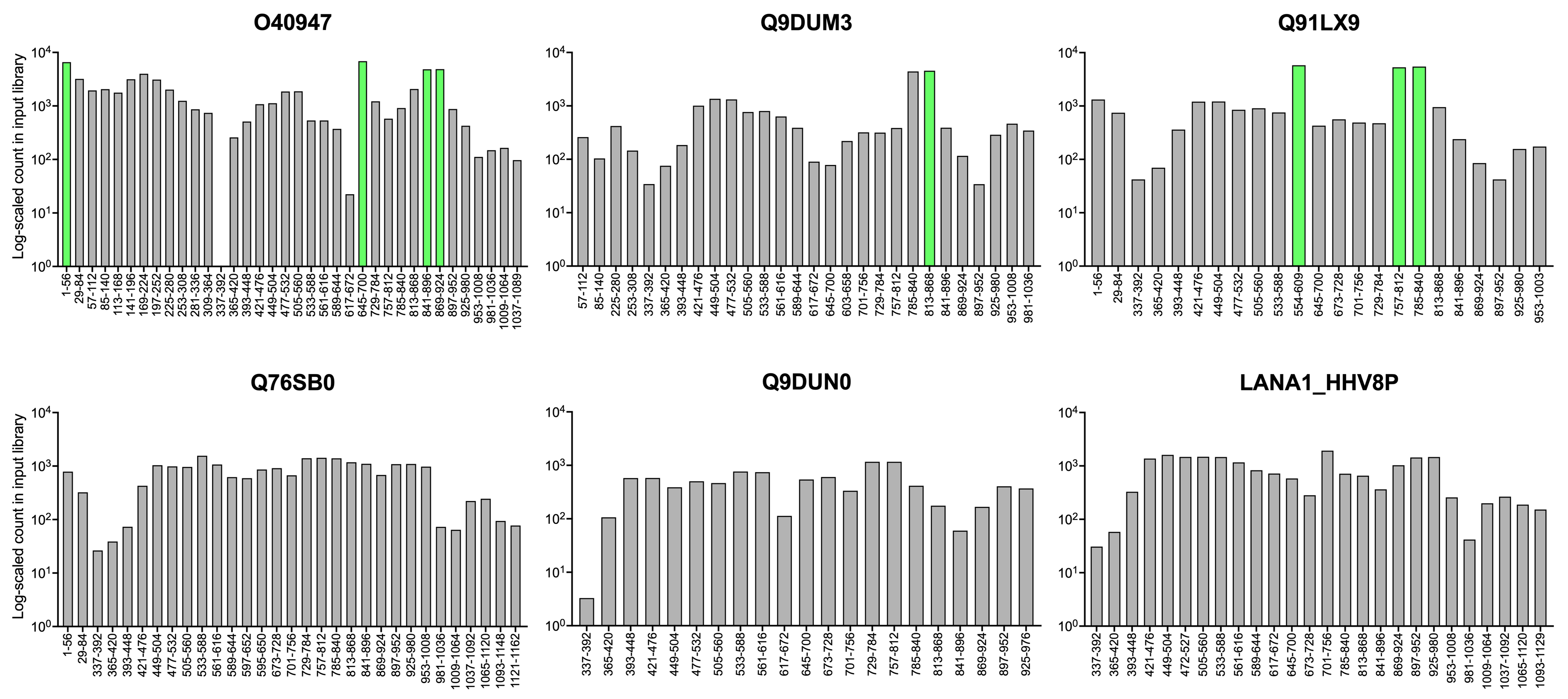

Supplement: S1 Fig — Each plot represents all the peptides from a UniProt entry, and each bar represents the log-scaled counts of a given peptide present in the library from the corresponding UniProt entry. The green bars represent the peptides filtered out due to significant binding in the mock-IPs. The missing peptides (no label on the x-axis) were non-unique amino acid sequences and were thus merged with the peptide that had the matching amino acid sequence. (TIF) [file ppat.1011033.s002.tif]

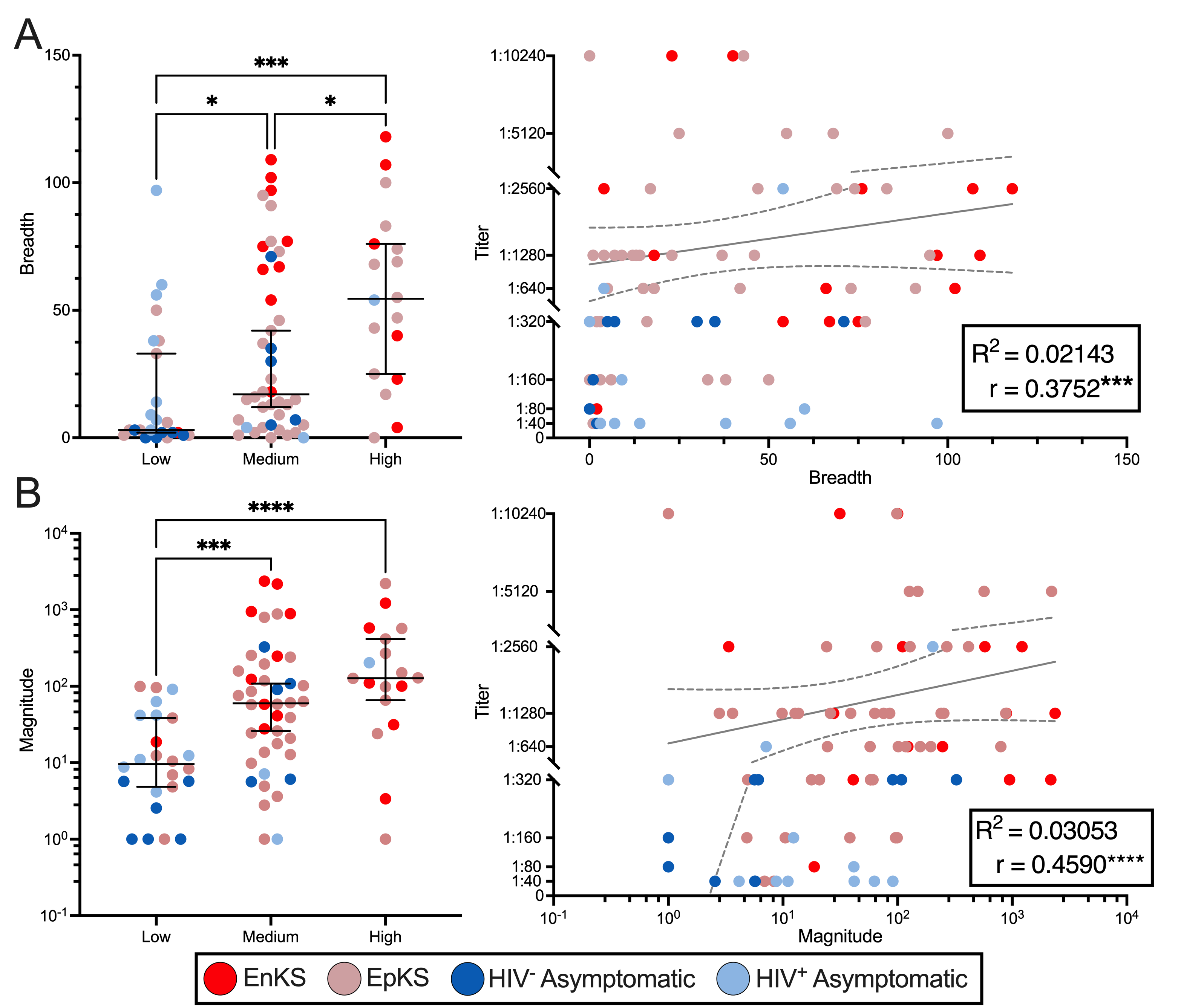

Supplement: S2 Fig — The KSHV Ab titer was determined using mIFA and categorized: low (1:40, 1:80, 1:160), medium (1:320, 1:640, 1:1280), and high (1:2560, 1:5120, 1:10240). (A) LANA Breadth, the number of reactive KSHV-LANA peptides per patient, was compared between titer levels (Kruskal-Wallis) and correlated with titer (Spearman). (B) LANA magnitude, the average magnitude (MLXP) of the reactive KSHV-LANA peptides per patient, was compared between titer levels (Kruskal-Wallis) and correlated with titer (Spearman). The 95% confidence interval of the linear regression is shown. Significance levels are indicated with asterisks, where p<0.05 *, p<0.01 **, p<0.001 ***, p<0.0001 ****. (TIF) [file ppat.1011033.s003.tif]

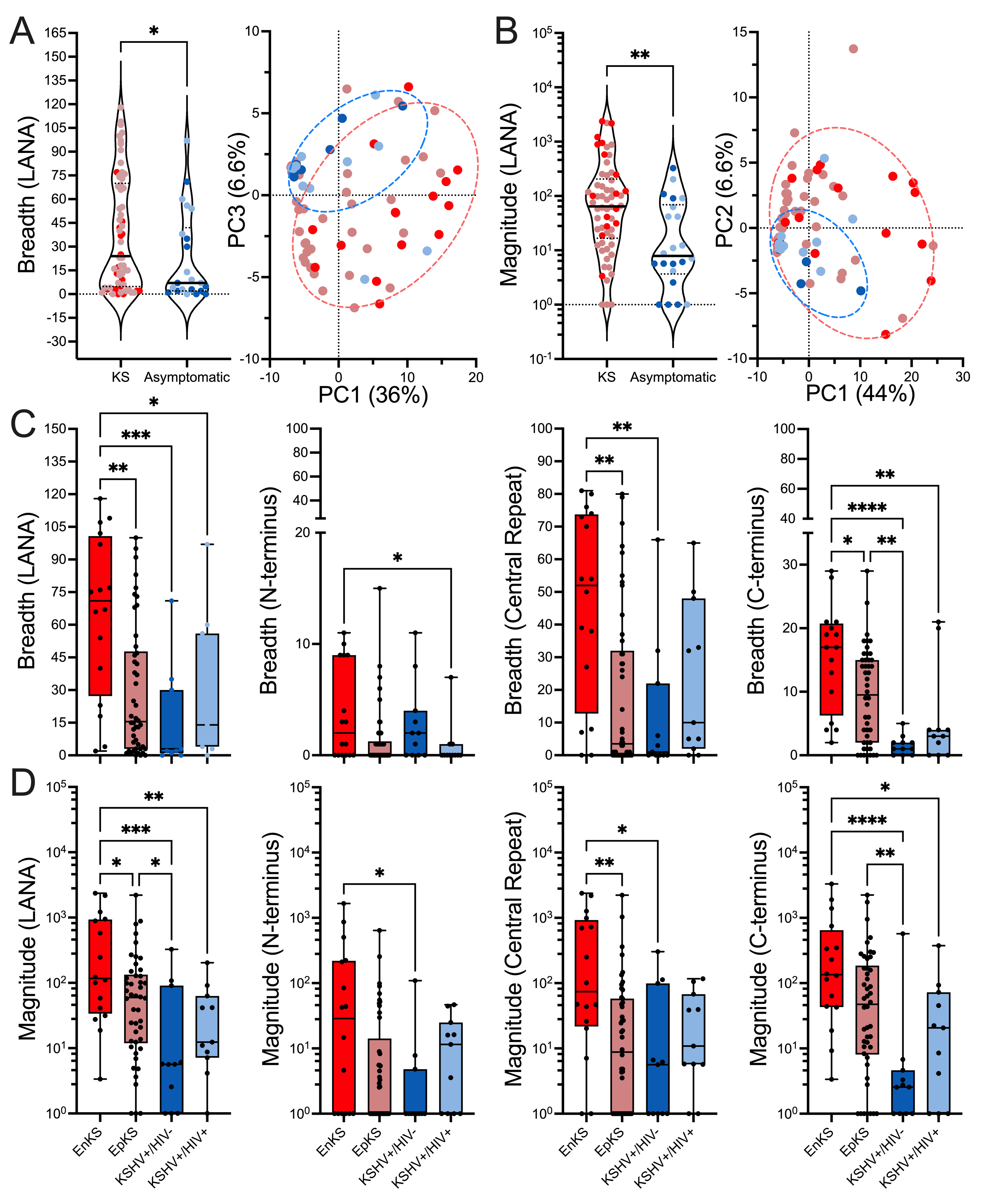

Supplement: S3 Fig — The number of reactive peptides and their magnitudes per individual was calculated and compared across disease states, in each group, and across each region within LANA, respectively. Comparison of (A) KSHV-LANA breadth and (B) magnitude across KS and asymptomatic individuals (Mann-Whitney) and 2D visualization of the PCA plot. Comparison of (C) KSHV-LANA breadth and (D) magnitude across EnKS, EpKS, KSHV+/HIV- and KSHV+/HIV+ individuals within each region (Kruskal-Wallis). Significance levels are indicated with asterisks, where p<0.05 *, p<0.01 **, p<0.001 ***, p<0.0001 ****. (TIF) [file ppat.1011033.s004.tif]

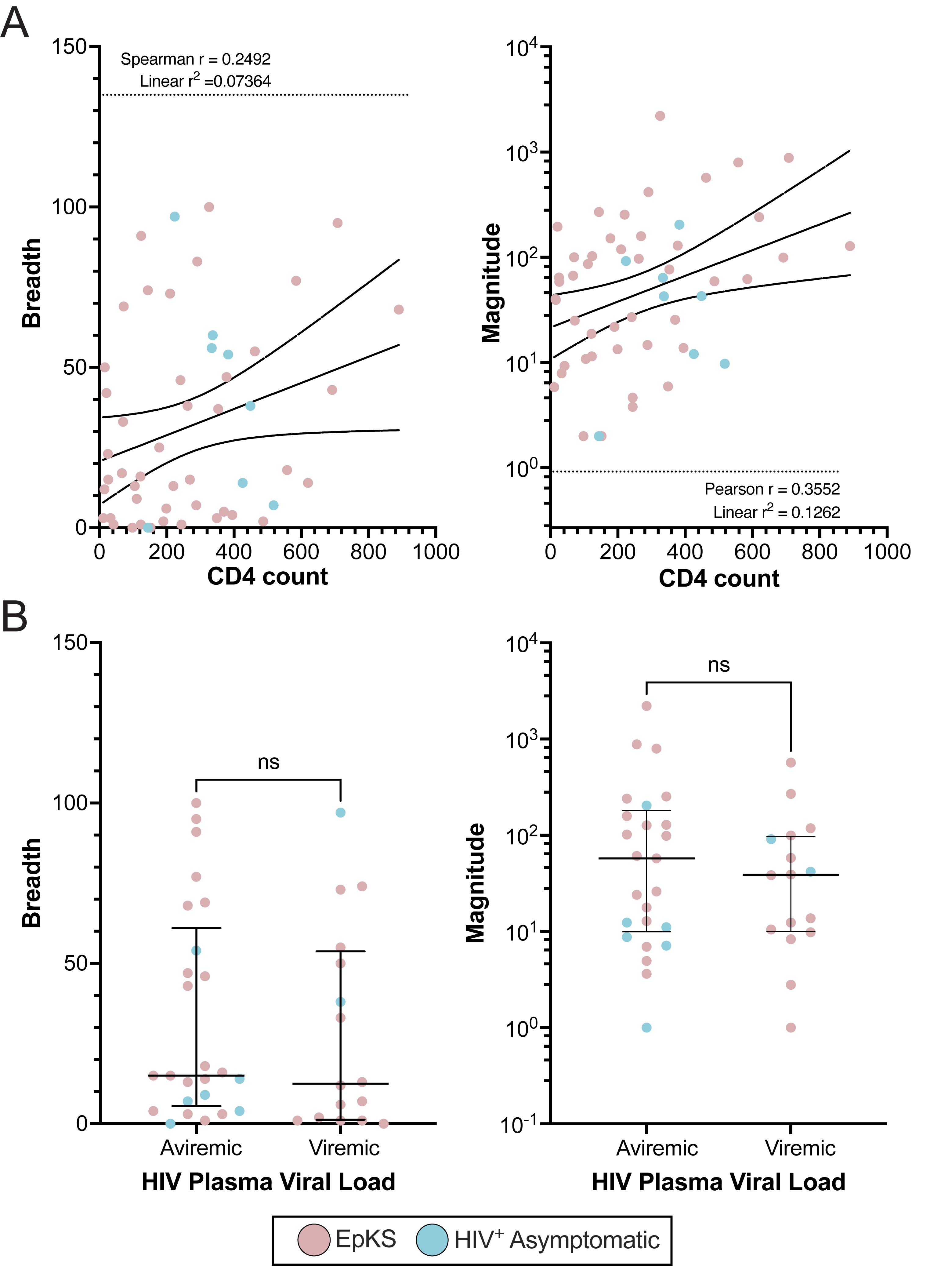

Supplement: S4 Fig — Both KSHV breadth and magnitude of Ab responses were tested for association with (A) CD4 count (n = 53) and (B) HIV viral load (n = 41). (TIF) [file ppat.1011033.s005.tif]
